# Bioinformatics Analysis of Publicly Available Single-Nuclei Transcriptomics Alzheimer’s Disease Datasets Reveals *APOE* Genotype-Specific Changes Across Cell Types in Two Brain Regions

**DOI:** 10.3389/fnagi.2022.749991

**Published:** 2022-04-27

**Authors:** Stella A. Belonwu, Yaqiao Li, Daniel G. Bunis, Arjun Arkal Rao, Caroline Warly Solsberg, Tomiko Oskotsky, Alice L. Taubes, Brian Grone, Kelly A. Zalocusky, Gabriela K. Fragiadakis, Yadong Huang, Marina Sirota

**Affiliations:** ^1^Bakar Computational Health Sciences Institute, University of California, San Francisco, San Francisco, CA, United States; ^2^Pharmaceutical Sciences and Pharmacogenomics Graduate Program, University of California, San Francisco, San Francisco, CA, United States; ^3^CoLabs, University of California, San Francisco, San Francisco, CA, United States; ^4^Bakar ImmunoX Initiative, University of California, San Francisco, San Francisco, CA, United States; ^5^Department of Pathology, University of California, San Francisco, San Francisco, CA, United States; ^6^Memory and Aging Center, University of California, San Francisco, San Francisco, CA, United States; ^7^Department of Neurology and Weill Institute for Neurosciences, University of California, San Francisco, San Francisco, CA, United States; ^8^Department of Pediatrics, University of California, San Francisco, San Francisco, CA, United States; ^9^Gladstone Institute of Neurological Disease, San Francisco, CA, United States; ^10^Division of Rheumatology, Department of Medicine, University of California, San Francisco, San Francisco, CA, United States; ^11^Department of Neurology, University of California, San Francisco, San Francisco, CA, United States

**Keywords:** Alzheimer’s disease, *APOE*, single-cell, RNA-sequencing, differential expression, network enrichment

## Abstract

Alzheimer’s Disease (AD) is a complex neurodegenerative disease that gravely affects patients and imposes an immense burden on caregivers. Apolipoprotein E4 (APOE4) has been identified as the most common genetic risk factor for AD, yet the molecular mechanisms connecting APOE4 to AD are not well understood. Past transcriptomic analyses in AD have revealed *APOE* genotype-specific transcriptomic differences; however, these differences have not been explored at a single-cell level. To elucidate more complex *APOE* genotype-specific disease-relevant changes masked by the bulk analysis, we leverage the first two single-nucleus RNA sequencing AD datasets from human brain samples, including nearly 55,000 cells from the prefrontal and entorhinal cortices. In each brain region, we performed a case versus control *APOE* genotype-stratified differential gene expression analysis and pathway network enrichment in astrocytes, microglia, neurons, oligodendrocytes, and oligodendrocyte progenitor cells. We observed more global transcriptomic changes in APOE4 positive AD cells and identified differences across *APOE* genotypes primarily in glial cell types. Our findings highlight the differential transcriptomic perturbations of APOE isoforms at a single-cell level in AD pathogenesis and have implications for precision medicine development in the diagnosis and treatment of AD.

## Introduction

Alzheimer’s disease (AD) is a heterogeneous neurodegenerative disorder, which accounts for at least 60% of dementia cases (Lane et al., 2018). Further underscoring the importance of AD research, cases of AD are projected to increase by more than threefold by 2050, yet there are currently no disease altering treatments (Hebert et al., 2013; Maresova et al., 2020). AD is defined by pathological hallmarks of aggregated extracellular amyloid-β (Aβ) plaques, and intracellular tau neurofibrillary tangles (Lane et al., 2018; Long and Holtzman, 2019). As a complex disease, AD has several environmental risk factors. Demographic risk factors include advanced age, low education level, and female sex. AD genetic risk factors such as Aβ precursor protein (*APP*), presenilin 1 (*PSEN1*), and presenilin 2 (*PSEN2*) point mutations lead to dominantly inherited early-onset AD and account for less than 1% of AD cases (Karch and Goate, 2015; Lane et al., 2018; Long and Holtzman, 2019).

The strongest genetic risk factor for late-onset or sporadic AD is the ε4 allele of the apolipoprotein E (*APOE*) gene. In humans, there are three common *APOE* allelic variants: ε2, ε3, and ε4, which differ based on single substitutions at amino acid residues 112 and 158. The ε3 allele is the most common variant, and is generally considered as a neutral form (Roses and Allen, 1996; Yu et al., 2014; Long and Holtzman, 2019). The ε2 allele is considered protective, and the ε4 allele is associated with increasing the risk of developing AD in a gene dose dependent manner (Long and Holtzman, 2019; Montagne et al., 2020). Specifically, one copy of the ε4 allele of *APOE* increases the risk of developing AD by three–fourfold, and two copies increases this risk by 12- to 15-fold (Karch and Goate, 2015; Long and Holtzman, 2019).

The APOE is a lipid-binding protein, which serves a central role in regulating lipid transport and metabolism. It is highly expressed in the liver and brain, where in the latter, it is primarily expressed in astrocytes (Yu et al., 2014; Long and Holtzman, 2019). APOE’s functionality in the central nervous system has implications for AD in both Aβ-dependent and Aβ-independent pathways. For instance, in addition to regulating Aβ clearance, APOE regulates lipoprotein metabolism, supports cell proliferation, repairs membranes, supports myelination, and maintains blood brain barrier (BBB) integrity (Mahley et al., 2006; Yu et al., 2014; Long and Holtzman, 2019). With regards to APOE isoforms, APOE4 has been linked to promoting Aβ retention by blocking its LRP1-mediated clearance (Mahley et al., 2006; Wan et al., 2020), insulin resistance through impaired insulin signaling (Zhao et al., 2017), BBB dysfunction and increased permeability (Zlokovic, 2011; Montagne et al., 2020), and regulating glycogen synthase kinase 3 (GSK3), a kinase highly involved in phosphorylation of tau (Hoe et al., 2006; Yu et al., 2014). Our study aims to identify transcriptomic differences associated with APOE isoforms at a single-cell level to better understand the underlying mechanisms contributing to AD pathophysiology and their specificity to each isoform. Transcriptomics represent a valuable means of understanding molecular underpinnings in disease conditions (Magistri et al., 2015; Allen et al., 2016; Wang et al., 2016; Patel et al., 2019; Wan et al., 2020; Neff et al., 2021); however, to our knowledge, in AD, APOE isoforms are yet to be investigated at a single-cell level, which can depict molecular profiles that would be otherwise masked in a bulk analysis.

In recent years, single-cell transcriptomic datasets were generated from the prefrontal (Mathys et al., 2019) and entorhinal (Grubman et al., 2019) cortices of human AD patients. First, Mathys et al. (2019) performed single-cell RNA sequencing (RNA-Seq) using prefrontal cortex samples from 24 individuals with high Aβ burden and related AD pathology, and 24 individuals with little to no Aβ burden or other pathologies. They observed distinct cell-type-specific perturbations mainly in myelination-related and protein homeostasis encoding genes. Second, Grubman et al. (2019) surveyed single-nucleus transcriptomes from the entorhinal cortices of 6 AD individuals and 6 cognitively normal controls. They identified repressed AD risk-associated gene expression patterns in the entorhinal cortex, such as transcription factor EB and regulator of lysosomal function, in astrocytes but upregulated in microglia. While both studies provided valuable human transcriptomic profiles at single-cell resolution and consistently reported cell type-variable *APOE* expression in AD, upregulated in microglia and downregulated in astrocytes, the authors did not examine cell type-specific gene expression differences in disease based on *APOE* genetic variants. In this study, we leverage these two publicly available datasets to study the cell type-specific transcriptomic effects of *APOE* genotype in AD across two brain regions: the prefrontal and entorhinal cortices. We aim to answer the following questions: (1) Which cell types are most affected at the transcriptomic level by *APOE* genotype in the context of AD? (2) What are the global and cell type-specific transcriptomic changes with respect to *APOE* genotype in the context of AD? and (3) Are there any transcriptomic changes that are specific to APOE4 that better explain AD pathophysiology?

## Materials and Methods

### Data and Code Availability

Single nuclei RNA-Seq (snRNA-seq) data and metadata were accessedfrom their respective repositories: the prefrontal cortex from the Accelerating Medicines Partnership Alzheimer’s Disease Project (AMP-AD) Knowledge Portal under the Religious Orders Study and Memory and Aging Project (ROSMAP)^1^,^2^, and the entorhinal cortex from a data repository provided^3^ by Grubman et al. (2019). Data from the entorhinal cortex may also be accessed from the Gene Expression Omnibus under the accession number GSE138852. Access to the prefrontal cortex dataset requires a formal request to ROSMAP. To enable other researchers to explore these datasets, all code necessary for recreating the reported analyses and figures within R, are available on Github at https://github.com/stebel5/AD_APOE_snRNAseq.

### Study Cohort Identification

**FIGURE 1:**
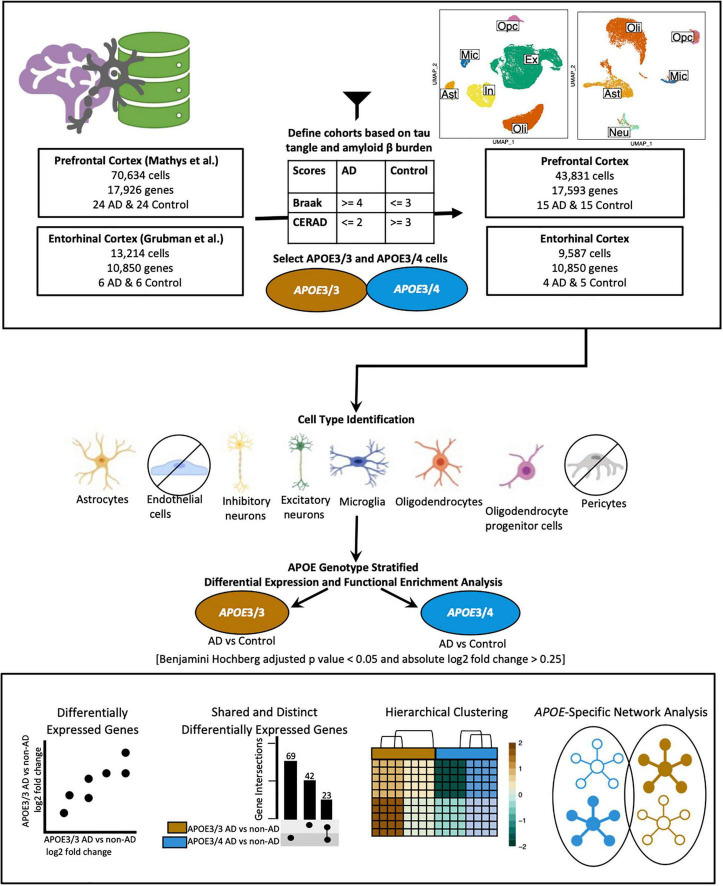
Workflow for sample definition and *APOE* genotype-stratified cell type-specific differential gene expression analysis and functional enrichment. AD and non-AD cells were determined based on tau tangle (Braak) and amyloid β plaque (CERAD) burden. Cell types were identified, and AD versus non-AD differential expression and pathway network enrichment analyses were performed separately for APOE3/3 and APOE3/4 cells.

We acquired publicly available snRNA-seq datasets from repositories specified by the first two single-cell transcriptomic AD studies (Grubman et al., 2019; Mathys et al., 2019). The prefrontal cortex dataset comprised 17,296 genes and 70,634 cells while the entorhinal cortex comprised 10,850 genes and 13,214 cells. Across both datasets, there was an overlap of 9,868 genes. Samples were classified based on tau neurofibrillary tangles, and Aβ plaque burden, using Braak clinical staging and Consortium to Establish a Registry for Alzheimer’s Disease (CERAD) scores, respectively (Mirra et al., 1991). Cases were identified as individuals with severe tau deposition (Braak stage ≥ 4) and high Aβ load (CERAD score ≤ 2), while non-AD controls were identified as individuals with low tau deposition (Braak stage ≤ 3) and low Aβ load (CERAD score ≥ 3). We also restricted our analysis to include samples with APOE3/3 (homozygous for allele ε3) and APOE3/4 (heterozygous ε3/ε4) genotypes due to the limited number of samples for relatively rare *APOE* genotypes (Figure 1).

The prefrontal cortex dataset initially consisted of age and sex matched samples from 48 individuals with varying levels of AD pathology. For the prefrontal cortex *APOE* genotype-stratified analysis, samples consisted of 14 APOE3/3 controls, 1 APOE3/4 control, 9 APOE3/3 cases and 8 APOE3/4 cases (Table 1).

**TABLE 1:** Prefrontal cortex cohort.

ID	Sex	*APOE*	Age	Diagnosis	Batch
ROS32	Female	3/3	90	AD	3
ROS27	Female	3/4	90	AD	11
ROS33	Female	3/3	90	AD	5
ROS36	Female	3/3	90	AD	8
ROS28	Female	3/3	87	AD	10
ROS29	Female	3/4	76	AD	3
ROS34	Female	3/4	74	AD	2
ROS39	Male	3/3	89	AD	5
ROS45	Male	3/4	89	AD	1
ROS42	Male	3/3	87	AD	10
ROS41	Male	3/4	85	AD	4
ROS48	Male	3/4	86	AD	9
ROS43	Male	3/3	83	AD	4
ROS37	Male	3/3	86	AD	2
ROS44	Male	3/3	80	AD	8
ROS10	Female	3/3	90	Control	11
ROS8	Female	3/3	87	Control	9
ROS9	Female	3/3	87	Control	10
ROS6	Female	3/3	83	Control	6
ROS12	Female	3/3	81	Control	7
ROS3	Female	3/3	79	Control	3
ROS18	Male	3/3	90	Control	5
ROS14	Male	3/4	88	Control	1
ROS23	Male	3/3	87	Control	12
ROS16	Male	3/3	84	Control	4
ROS19	Male	3/3	80	Control	8
ROS13	Male	3/3	80	Control	1
ROS20	Male	3/3	80	Control	9
ROS15	Male	3/3	79	Control	2
ROS17	Male	3/3	76	Control	4

The entorhinal cortex dataset initially consisted of age and sex matched samples from 6 AD and 6 control subjects, as indicated by Grubman et al. (2019). Cases were noted to have a history of AD, while controls had no history of AD or cognitive impairment, as reported by treating general practitioners. Braak scores were provided only for cases, while clinical history and amyloid pathology records were provided for all subjects. Amyloid pathology information was provided using the categories: “Numerous diffuse and neuritic Aβ plaque,” “Occasional diffuse plaque in cortex,” and “None.” Using criteria from the Rush Alzheimer’s Disease Center clinical codebook provided with the prefrontal cortex dataset, we converted these measures of neuritic plaques into CERAD scores of 1 (Definite), 3 (Possible), and 4 (No AD), respectively. This allowed us to systematically identify cases and controls in both datasets using the same criteria. For the entorhinal cortex *APOE* genotype-stratified analysis, samples consisted of 4 cases, and 5 controls (Table 2). Three of the cases were from APOE3/4 individuals, while one was from an APOE3/3 individual, and of the controls, four were from APOE3/3 individuals and the one was from an APOE3/4 individual.

**TABLE 2:** Entorhinal cortex cohort.

ID	Sex	*APOE*	Age	Diagnosis	Batch
AD1	Male	3/4	91	AD	AD1_AD2
AD2	Male	3/4	83.8	AD	AD1_AD2
AD4	Female	3/3	83.0	AD	AD3_AD4
AD6	Male	3/4	74.6	AD	AD5_AD6
Ct1	Female	3/3	67.3	Control	Ct1_Ct2
Ct2	Female	3/3	82.7	Control	Ct1_Ct2
Ct3	Male	3/3	72.6	Control	Ct3_Ct4
Ct4	Male	3/4	75.6	Control	Ct3_Ct4
Ct5	Male	3/3	77.5	Control	Ct5_Ct6

### Single Cell Data Processing, Cell Type Identification, and Batch Correction

All data processing was conducted separately for each dataset with (R Core Team, 2020) version 4.0.0 (2020-04-24) using RStudio (R Studio Team, 2020), using Seurat (Stuart et al., 2019) (v3.1.5). We generated visualizations using BioRender^4^ (Figure 1), dittoSeq (Bunis et al., 2020) (v1.0.2), an R package for analysis and color blind friendly visualization of single-cell and bulk RNA-Seq data, ggplot2 (Wickham, 2009), and UpsetR (Conway et al., 2017).

#### Prefrontal Cortex

We downloaded a filtered raw expression matrix of 17,296 genes and 70,634 cells from the prefrontal cortex from the AMP-AD Knowledge Portal and used Seurat’s *Read10x* function to generate a count data matrix using the raw count matrix, cell names, and barcodes files provided. A Seurat object was created with the count data matrix and metadata, keeping genes present in at least 3 cells, and cells meeting cohort selection criteria with at least 200 genes. Additionally, we selected samples from APOE3/3 and APOE3/4 individuals (Table 1), which resulted in a dataset with 43,831 cells (Supplementary Table 1) and 17,593 genes. Log normalization was performed with a *scale.factor* of 10,000, and *FindVariableFeatures* was run using 3,188 features, as specified in the original paper. The data matrix was then scaled with “nCount_RNA” regressed out, and dimensionality reduction was performed with the appropriate dimensions selected based on the corresponding Principal component analysis (PCA) elbow plot. Dimensionality reduction confirmed that there were no batch effects present (Supplementary Figure 1). As we found the original paper’s cell type identification to be comprehensive, we kept the cell type labels for the further analysis (Supplementary Table 1). Due to low cell counts, we did not analyze pericytes and endothelial cells.

#### Entorhinal Cortex

A filtered raw expression matrix of 10,850 genes and 13,214 cells from the entorhinal cortex was downloaded from a data repository provided by Grubman et al. (2019). Originally composed of 33,694 genes and 14,876 cells, genes and cells were filtered as described by Grubman et al. (2019). Cells from APOE3/3 and APOE3/4 individuals were selected (Table 2), and a Seurat object was created to consist of genes in at least 3 cells, and cells with at least 200 genes. Normalization was performed using Seurat’s *SCTransform* method, and Seurat’s integration workflow (Stuart et al., 2019) was performed to correct the confounded batches introduced by the experimental design. In this dataset, as shown in Table 2, control samples were processed separately from cases, male samples were processed separately from female samples, and all but one batch contained one *APOE* genotype. Dimensionality reduction was performed using values from the integrated assay to assess successful batch correction (Supplementary Figure 1).

To identify cell types, we adopted techniques from the original paper. Briefly, Grubman et al. (2019) used Seurat’s *AddModuleScore* function to calculate association scores using lists of brain cell type markers of an unspecified number from the BRETIGEA (McKenzie et al., 2018) package. They labeled cells based on which set of markers they had the highest score for, identified hybrids as cells where the highest and second highest score were within 20% of each other, and relabeled unidentified cells based on *z*-score transformation of the gene score distribution. In our case, we used lists of 200 genes for astrocytes, neurons, microglia, oligodendrocytes, oligodendrocyte progenitor cells (OPCs), and endothelial cells to label cells and hybrids to exclude as defined by Grubman et al. (2019). We further confirmed successful cell type identification by visualizing scores in a feature plot and assessing homogeneity and separation of clusters in PCA, and Uniform Manifold Approximation and Projection (UMAP) plots based on principal components and expression of top marker genes across cell types. Due to limitations in the number of cells, we excluded endothelial cells from further analyses, which resulted in a dataset comprising 10,850 genes and 9,587 cells (Supplementary Table 2).

### Cell Type-Specific *APOE* Genotype-Stratified Differential Expression Analysis

To generate transcriptomic disease signatures relative to *APOE* genotype in each cell type, we used Limma-Voom (Law et al., 2014; Ritchie et al., 2015; Phipson et al., 2016). We included the risk factor sex as a covariate in our design formula for both datasets. In the entorhinal cortex dataset, sex, instead of batch, also accounted for the confounding relationships introduced by the original study design, allowed for an appropriate model fit, and avoided the collinearity limitation observed with including batch in the design. Additionally, as samples were age matched, we also did not include age in our design formula. A *DGEList* object was then created from a matrix of counts extracted from the corresponding Seurat objects. To improve the accuracy of mean-variance trend modeling and lower the severity of multiple testing correction, lowly expressed genes were filtered out using edgeR’s *FilterByExpr* with default parameters. Normalization was performed with Trimmed Mean of *M*-values with singleton pairing (TMMwsp), followed by voom, model fitting with a contrast matrix of each case-control comparison for each cell type-*APOE* group, and Empirical Bayes fitting of standard errors. We performed a cell type-specific AD versus control gene expression comparison in each *APOE* variant group separately in our defined prefrontal cortex cohort, entorhinal cohort, and male-only prefrontal cortex cohort, in which we excluded sex as a covariate. Differentially expressed genes (DEGs) were selected using a Benjamini-Hochberg (BH) corrected *p*-value less than 0.05, and an absolute log base twofold change (log2 FC) greater than 0.25, meaning greater than 20% change in expression. We visualized unique and shared disease related gene expression changes in cell types of each *APOE* genotype using pairwise and violin plots of gene expression, hierarchical clustering of samples using AD compared to control pseudobulk cell type gene expression, and Upset plots, where genes with more overlaps across the groups compared were prioritized for labeling.

### Functional Enrichment Analysis and Network Visualization

We performed an overrepresentation analysis of DEGs from the cell type-specific *APOE* genotype-stratified analysis of cells from the prefrontal and entorhinal cortex using gprofiler (Raudvere et al., 2019), a web tool for functional enrichment using an input gene list. We queried DEGs comparison split by upregulated and downregulated expression to identify enriched pathways. In addition to Gene Ontology, we include pathways from KEGG Reactome and WikiPathways; miRNA targets from miRTarBase and regulatory motif matches from TRANSFAC; tissue specificity from Human Protein Atlas; protein complexes from CORUM, and human disease phenotypes from Human Phenotype Ontology. We followed a previously established protocol (Reimand et al., 2019) for network enrichment analysis on pathway results derived from our cell type-specific DEGs. Briefly, pathway results were imported into the Cytoscape visualization application, EnrichmentMap. We collapsed redundant and related pathways into single biological themes and further filtered significant pathways using a BH adjusted *p*-value < 0.01. Individual biological themes were defined and summarized using the AutoAnnotate Cytoscape application.

## Results

### Sample Classification and Analytic Workflow

We classified samples into AD and control groups based on tau tangle and Aβ plaque burdens, using Braak clinical staging and CERAD scores (Mirra et al., 1991), respectively (AD: Braak stage ≥ 4, CERAD score ≤ 2; Control: Braak stage ≤ 3, CERAD score ≥ 3) (Figure 1). Next, from the prefrontal cortex cohort (Table 1), we analyzed snRNA-seq data containing 43,831 cells (Supplementary Table 1) and 17,593 genes, and from the entorhinal cortex cohort (Table 2), we analyzed snRNA-seq data containing 9,587 cells (Supplementary Table 2) and 10,850 genes. Both datasets were acquired from different sets of individuals.

Due to the limited number of samples for relatively rare *APOE* genotypes, we focused our analysis on comparisons between AD and non-AD groups with APOE3/3 (homozygous for allele ε3) and APOE3/4 (heterozygous ε3/ε4) genotypes. We performed an *APOE* genotype-stratified differential gene expression (DGE) analysis comparing age-matched AD cases to controls, with sex as a covariate, in excitatory (Ex) and inhibitory (In) neurons for the prefrontal cortex specifically, undistinguished neurons (Neu) for the entorhinal cortex, and astrocytes (Ast), microglia (Mic), oligodendrocytes (Oli), and OPCs for both cohorts (Supplementary Figures 1, 2). DEGs were selected using cutoffs of a BH adjusted *p*-value < 0.05 and >20% change in expression. DEGs were further passed as inputs to identify pathways for subsequent network analysis. We examined gene expression and network changes in AD compared to non-AD samples to identify cell type-specific and shared changes based on *APOE* genotype (Figure 1).

**FIGURE 2:**
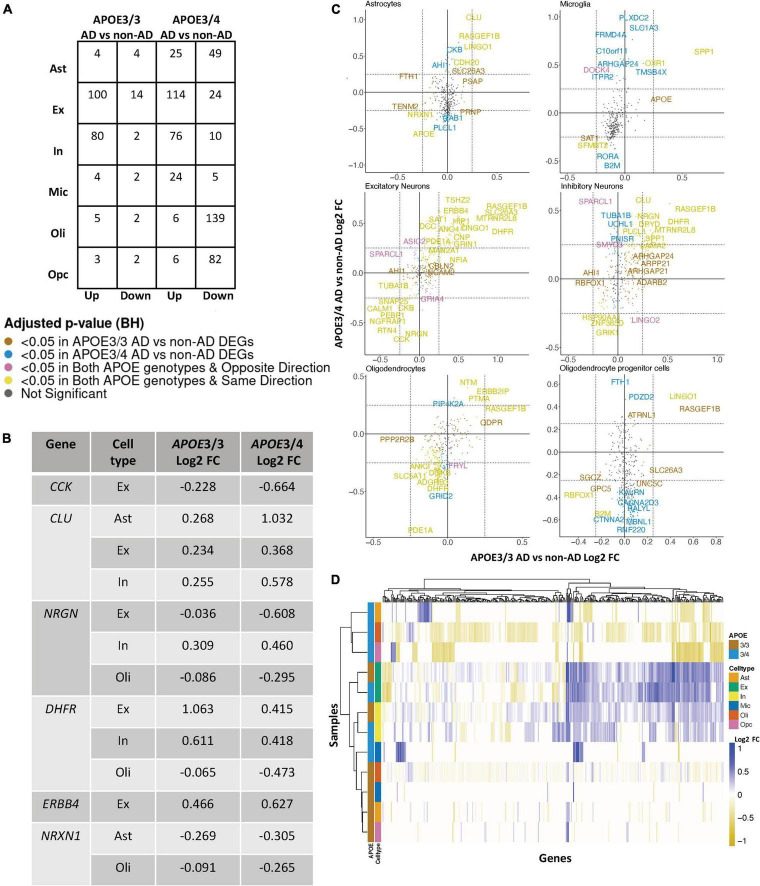
*APOE* genotype-stratified cell type-specific disease signatures in the prefrontal cortex. **(A)** AD versus non-AD DEG counts for astrocytes (Ast), excitatory (Ex) and inhibitory (In) neurons, microglia (Mic), oligodendrocytes (Oli), and oligodendrocyte progenitor cells (Opc) in surveyed *APOE* genotypes. DEGs were selected using a BH adjusted *p*-value < 0.05 and >20% change in expression. **(B)** Subset of DEGs shared by both *APOE* genotypes and their corresponding change in expression. **(C)** Pairwise DEG plots of DEGs in APOE3/3 and APOE3/4 samples using change in expression. Genes shown are significant and have >20% change in expression in at least one *APOE* genotype. Colors indicate significance level of DEGs and whether DEGs are unique or shared by *APOE* genotypes. **(D)** Change in expression of all genes in the DE analysis clustered by cell type and *APOE* genotype.

### *APOE* Genotype-Stratified Differential Gene Expression Analysis in the Prefrontal Cortex Identifies Distinct Alzheimer’s Disease-Related Changes in Astrocytes, Oligodendrocytes, and Oligodendrocyte Progenitor Cells

Leveraging data from Mathys et al. (2019), we identified DEGs in all cell type and *APOE* genotype pairings when comparing AD to control tissue from 43,831 cells covering 17,593 genes (Supplementary Figure 2). Interestingly, DEGs were primarily downregulated in APOE3/4 astrocytes, oligodendrocytes and OPCs, while they were primarily upregulated in both APOE3/3 and APOE3/4 neurons (Figure 2A). Altogether, across all cell types we identified 278 unique DEGs (Supplementary Table 3). Of the 278 DEGs, 8 were specific to APOE3/3 and 135 were specific to APOE3/4. We observed DEGs previously linked to AD [*CLU* (Kok et al., 2011; Karch and Goate, 2015), *CCK* (Mazurek and Beal, 1991; Chen et al., 2019; Plagman et al., 2019), *NRGN* (Thorsell et al., 2010; Jin et al., 2013), *DHFR* (Cario et al., 2011; Philip et al., 2015), *ERBB4* (Mitchell et al., 2013; Mouton-Liger et al., 2020), *NRXN1* (Mozhui et al., 2011)], which were shared by APOE3/3 and APOE3/4 cells. In most cases, expression differences in these genes were in the same direction across genotypes, but with greater fold changes in APOE3/4 as compared to APOE3/3 cells (Figure 2B). Across cell types, while the majority of DEGs were shared and in consistent direction across APOE3/3 and APOE3/4 cells (Figure 2C, yellow color and Supplementary Figure 2), there were a few shared DEGs with opposite directionality of expression changes, such as *DOCK4* in microglia, *SPARCL1* in neurons, and *FRYL* in oligodendrocytes (Figure 2C, pink colorand Supplementary Figure 2).

**FIGURE 3:**
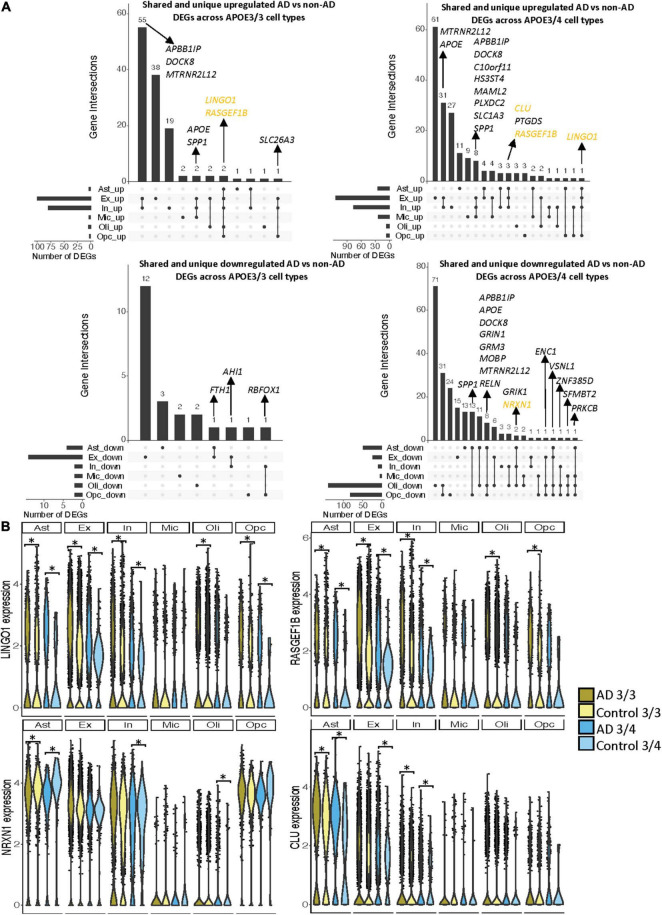
Shared and unique disease signatures across cell types in APOE3/3 and APOE3/4 prefrontal cortex samples. **(A)** Upset plots indicating intersections of AD versus non-AD DEGs (BH adjusted *p*-value < 0.05 and >20% change in expression) across cell types. Rows correspond to cell types. The bar chart shows the number of single and common sets of DEGs across cell types. Single filled dots represent a unique set of DEGs for each cell type. Multiple filled black dots connected by vertical lines represent common sets of DEGs across cell types. DEGs with more overlaps across groups were prioritized for labeling. **(B)** *LINGO1*, *RASGEF1B*, *NRXN1* and *CLU* expression. Asterisks represent meeting both significance (BH adjusted *p*-value < 0.05) and change in expression (>20%) thresholds. Colors correspond to *APOE* genotype and AD status.

Notably, some DEGs in AD patients relative to controls were shared across multiple cell types (Figure 3A). Examples of some DEGs in AD patients relative to controls that overlap most across cell types within or across *APOE* genotypes include APP binding family B member 1 interacting protein (*APBB1IP*), and *DOCK8*, a protein highly involved in brain development and immune response (Nishikimi et al., 2013). Both were differentially expressed in most APOE3/4 cell types and in APOE3/3 neurons (Figure 3A). Interestingly, for both *APBB1IP* and *DOCK8*, we observed cell type-specific effects. Both genes were downregulated in astrocytes, oligodendrocytes and OPCs and upregulated in microglia and neurons from APOE3/4 AD patients versus APOE3/4 controls. In APOE3/3 individuals, both genes were only significantly upregulated in neurons in AD patients versus controls. *APOE* itself was also differentially expressed in AD patients versus controls, with an increase in both APOE3/4 and APOE3/3 neurons and in APOE3/3 microglia as well as a decrease in APOE3/4 astrocytes, oligodendrocytes, and OPCs. *MTRNR2L12* expression, which encodes a humanin isoform necessary for neuroprotection and anti-apoptotic function suggested to have utility as a blood marker for cognitive disability and early dementia for adults with Down Syndrome (Bik-Multanowski et al., 2015; Mahajan et al., 2018), was very similar to *APOE* expression. Hierarchical clustering of samples using AD compared to control pseudobulk cell type gene expression (Figure 2D) showed samples to cluster by *APOE* genotype before cell type identity for all cell types except neurons. Generally, through our *APOE* genotype-stratified analysis we observed more similarities in AD versus control DEGs across *APOE* genotypes in neuronal populations (both excitatory and inhibitory neurons), and differences primarily in non-neuronal cells (astrocytes, oligodendrocytes, and OPCs) (Figure 3A). In addition to identifying shared DEGs across cell types and *APOE* genotypes, we also observed a larger range of expression change in the analysis of APOE3/4 AD versus control (−0.834, 1.032; median = −0.273) compared to the analysis of APOE3/3 AD versus control (−0.503,1.115; median = 0.342), which we visualized in a few shared DEGs such as *LINGO1*, *NRXN1*, *RASGEF1B*, and *CLU* (Figure 3B).

As the prefrontal cohort contained a sole non-AD sample with the APOE3/4 genotype from a male donor, we performed a sensitivity analysis in male samples to determine whether similar gene signatures remain. We identified 300 unique DEGs across all cell types (Supplementary Figure 3 and Supplementary Table 4). Of these DEGs, 18 were specific to APOE3/3 cells and 128 to APOE3/4 cells. Like the previous analysis, we observed more differences in perturbed gene profiles across *APOE* genotypes in astrocytes, oligodendrocytes, and OPCs, where DEGs were primarily downregulated in APOE3/4 cells. Additionally, clustering samples using AD compared to control pseudobulk cell type gene expression also showed a stronger clustering by *APOE* genotype than cell type identity (Supplementary Figure 3).

### *APOE* Genotype-Stratified Differential Gene Expression Analysis in the Entorhinal Cortex Identifies Distinct Alzheimer’s Disease-Related Changes in Microglia and Oligodendrocytes

**FIGURE 4:**
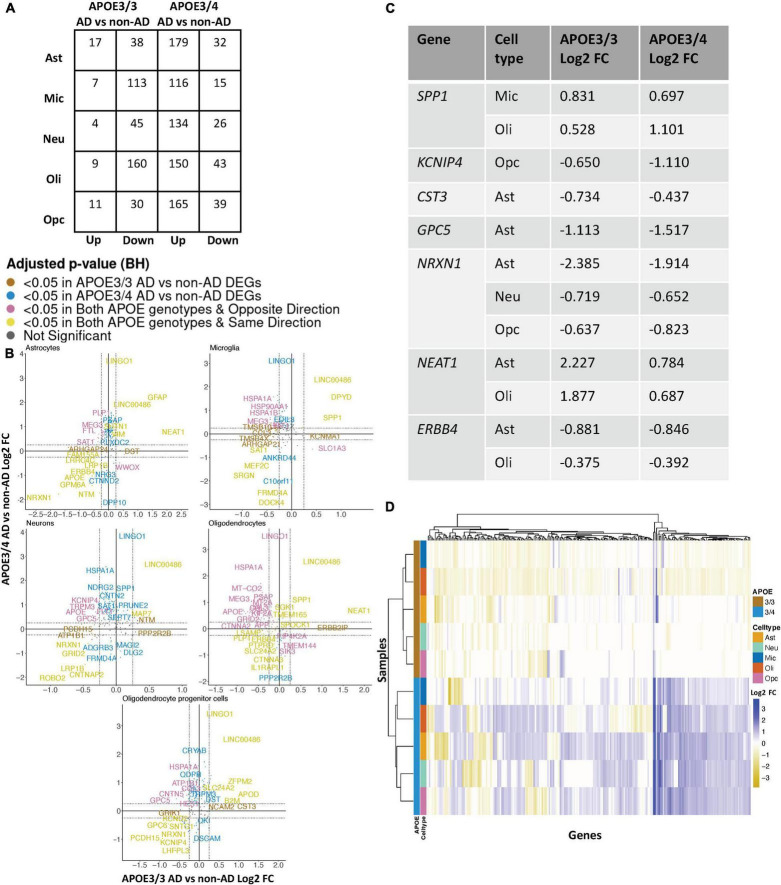
*APOE* genotype-stratified cell type-specific disease signatures in the entorhinal cortex. **(A)** AD versus non-AD DEG counts for astrocytes (Ast), neurons (Neu), microglia (Mic), oligodendrocytes (Oli), and oligodendrocyte progenitor cells (Opc) in surveyed *APOE* genotypes. DEGs were selected using a BH adjusted *p*-value < 0.05 and >20% change in expression. **(B)** Pairwise DEG plots of DEGs in APOE3/3 and APOE3/4 samples using change in expression. Genes shown are significant and have >20% change in expression in at least one *APOE* genotype. Colors indicate significance level of DEGs and whether DEGs are unique or shared by *APOE* genotypes. **(C)** Subset of DEGs shared by both *APOE* genotypes and their corresponding change in expression. **(D)** Change in expression of all genes in the DE analysis clustered by cell type and *APOE* genotype.

Leveraging data from Grubman et al. (2019), we identified DEGs in all cell type and *APOE* genotype pairings when comparing AD to control tissue from 9,587 cells and 10,850 genes. We found DEGs to be primarily downregulated in APOE3/3 AD versus control and upregulated in APOE3/4 AD versus control (Figure 4A). Altogether, across all cell types we identified 232 unique DEGs (Supplementary Table 5). Of the DEGs, 29 were specific to the APOE3/4 AD, and none were specific to the APOE3/3 AD. In each cell type, we observed more DEGs in the APOE3/4 comparison, some of which were shared with the APOE3/3 analysis, though often with consistent opposite directionality [Figure 4B; yellow (same) and pink (opposite) colors and Supplementary Figure 2]. For DEGs shared across *APOE* genotypes with consistent directionality, we observed differences in fold changes (Figure 4C). We also observed a higher proportion of common DEGs across *APOE* groups in microglia and oligodendrocytes than in other cell types, and in most cases, there was opposite directionality of gene expression changes between the APOE3/3 AD versus control comparison and APOE3/4 AD versus control comparison. Overall, clustering samples using AD compared to control pseudobulk cell type gene expression (Figure 4D) showed consistent clustering of samples by *APOE* genotype.

**FIGURE 5:**
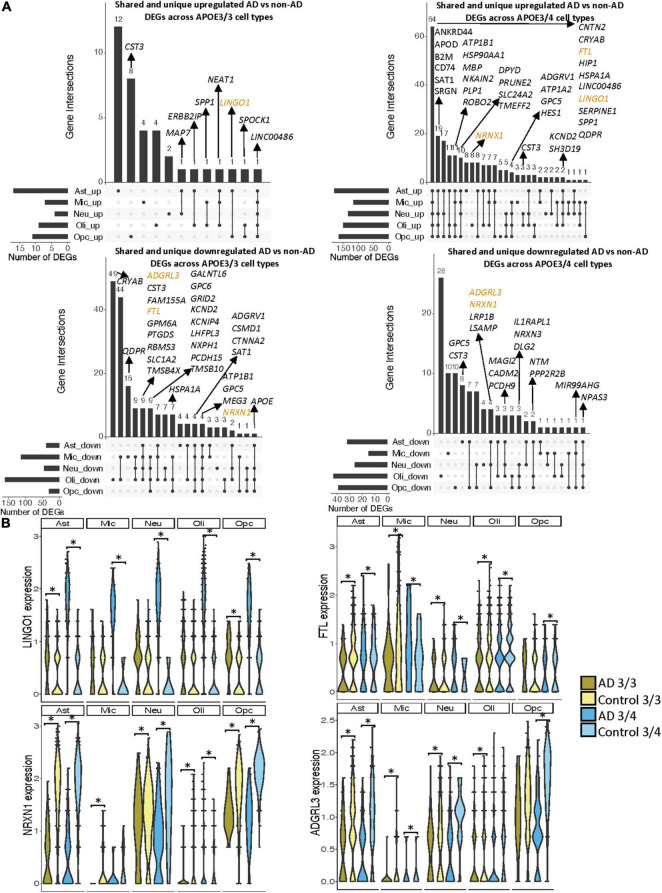
Shared and unique disease signatures across cell types in APOE3/3 and APOE3/4 entorhinal cortex samples. **(A)** Upset plots indicating intersections of AD versus non-AD DEGs (BH adjusted *p*-value < 0.05 and >20% change in expression) across cell types. Rows correspond to cell types. The bar chart shows the number of single and common sets of DEGs across cell types. Single filled dots represent a unique set of DEGs for each cell type. Multiple filled black dots connected by vertical lines represent common sets of DEGs across cell types. A subset of DEGs shared are highlighted to show examples of shared genes. **(B)** *LINGO1*, *FTL*, *NRXN1*, and *ADGRL3* expression. Asterisks represent meeting both significance (BH adjusted *p*-value < 0.05) and change in expression (>20%) thresholds. Colors represent *APOE* genotype and diagnosis.

When surveying DEG overlaps across cell types in the entorhinal cortex, consistent with the prefrontal cortex analysis, we observed more DEGs in AD patients relative to controls shared across APOE3/4 cell types than across APOE3/3 cell types (Figure 5A). To highlight some of these DEGs that overlap most across cell types, in the APOE3/3 case-control comparisons, six DEGs –*ATP1B1* (Wen et al., 2018), a sodium and potassium ATPase necessary for regulating ionic gradients; *CST3* (Hua et al., 2012), an AD risk factor; GPC5 (Shin et al., 2013), *a* neurotrophic factor; *MEG3* (Zhou et al., 2012), a long non-coding RNA and apoptosis regulator*; NRXN1*; and *LINC00486*, a relatively uncharacterized long non-coding transcript – were shared by all cell types. *LINC00486* was upregulated in all APOE3/3 cell types in AD, *ATP1B1, GPC5, MEG3, and NRXN1* were downregulated in all APOE3/3 cell types in AD, and *CST3* was downregulated in all APOE3/3 cell types in AD, except OPCs where it was upregulated. These DEG’s were also reflected in APOE3/4 cells, with *LINC00486* upregulated in all cell types, *ATP1B1 and MEG3* upregulated in non-neuronal cell types, *NRXN1* upregulated in oligodendrocytes and downregulated in all other cell types, *GPC5* downregulated in astrocytes and upregulated in all other cell types, and *CST3* downregulated in astrocytes and upregulated in neurons and oligodendrocytes in case-control comparisons.

Overall, in the APOE3/4 case-control comparisons, 87 DEGs were shared in all cell types, with 64 consistently upregulated in AD tissue and 23 with mixed directionality across cell types when comparing AD to control tissue (Figure 5A). Of these shared DEGs, a few with higher expression changes between AD and controls include *MBP*, a gene important for myelination (Koenning et al., 2012; Ferrer and Andrés-Benito, 2020) that was upregulated in all APOE3/4 cell types in AD except oligodendrocytes, and *LINGO1*, which was upregulated in all APOE3/4 cell types as well as APOE3/3 astrocytes and OPCs in AD. Interestingly the average expression change for *LINGO1* in APOE3/4 AD samples (3.52) was much higher than that of the APOE3/3 AD samples (0.451). Additionally, protein folding *HSPA1A*, the neuroprotective chaperone and apoptosis regulator *CRYAB* (Ousman et al., 2007), and quinoid dihydropteridine reductase (*QDPR*) were upregulated in all APOE3/4 cell types in AD. However, *HSPA1A* was downregulated in APOE3/3 microglia, oligodendrocytes, and OPCs, *CRYAB* was downregulated in APOE3/3 oligodendrocytes, and *QDPR* was downregulated in APOE3/3 microglia in AD. The latter two genes have previously been observed to be upregulated in oligodendrocytes and OPCs of pathologically confirmed AD individuals (Mathys et al., 2019), most of them are usually APOE4 carriers. We also observed a larger range of case-control expression change in APOE3/4 cells (−2.918, 3.839; median = 0.688) compared to APOE3/3 cells (−2.385, 2.227; median = −0.436), which we visualized in a few shared DEGs such as *LINGO1*, *NRXN1*, *FTL*, and *ADGRL3* (Figure 5B). Largely, when comparing AD to non-AD cells in the entorhinal cortex, while we observed changes relevant to AD pathophysiology across APOE3/3 and APOE3/4 genotypes, we also observed flipped DEG expression profiles across both *APOE* genotypes primarily in non-neuronal cells, and more universal transcriptional changes and changes of higher amplitude in the APOE3/4 AD versus control comparison as compared to APOE3/3 AD versus control comparison.

### Comparative Analysis Across Brain Regions Shows More Alzheimer’s Disease-Related Transcriptomic Changes in the Entorhinal Cortex Compared to the Prefrontal Cortex, With Consistent *APOE* Genotype-Specific Disease Signatures

**FIGURE 6:**
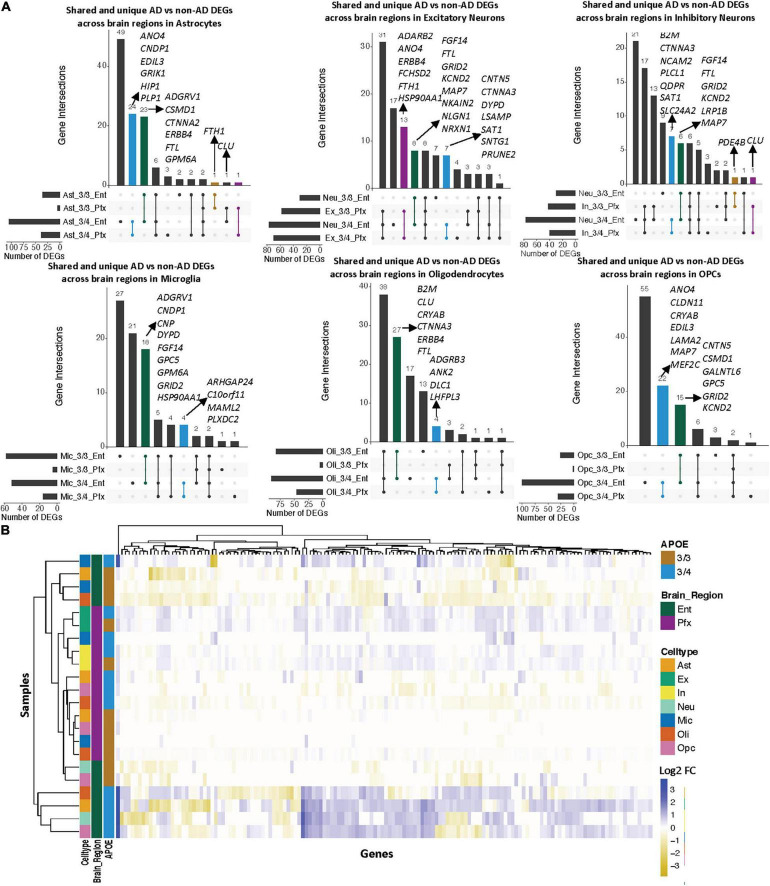
*APOE* genotype-stratified cell type-specific disease signatures across brain regions. **(A)** Upset plots indicating intersections of AD versus non-AD DEGs (BH adjusted *p*-value < 0.05 and >20% change in expression) within cell types across brain region and *APOE* genotype. Rows correspond to brain region and *APOE* genotype pairings. The bar chart shows the number of single and common sets of DEGs across brain regions and *APOE* genotype pairings. Single filled dots represent a unique set of DEGs for each brain region and *APOE* genotype pairing. Multiple filled black dots connected by vertical lines represent common sets of DEGs across brain region and *APOE* genotype pairings. Bar chart colors correspond to whether DEGs are shared between brain regions or *APOE* genotype using the bottom right key. DEGs with more overlaps across groups were prioritized for labeling. **(B)** Change in expression of all genes in the DE analysis of both brain regions clustered by cell type, brain region, and *APOE* genotype.

We observed a higher number of DEGs and larger expression change magnitudes across cell types in the entorhinal cortex than in the prefrontal cortex in AD. The number of shared DEGs within cell types across *APOE* genotype groups was highest in the entorhinal cortex in AD, while the number of shared DEGs within cell types across brain regions was highest in APOE3/4 cells in AD (Figure 6A). With hierarchical clustering of per-cell and genotype group pseudobulk expression, while we do not observe total clustering by any of the variables examined, we see some clustering by brain region, and within these brain regions, by *APOE* genotype (Figure 6B).

### Pathway and Network Analysis Reveal *APOE* Genotype-Specific Perturbed Biological Processes Primarily in Glial Cells Across Brain Regions

**FIGURE 7:**
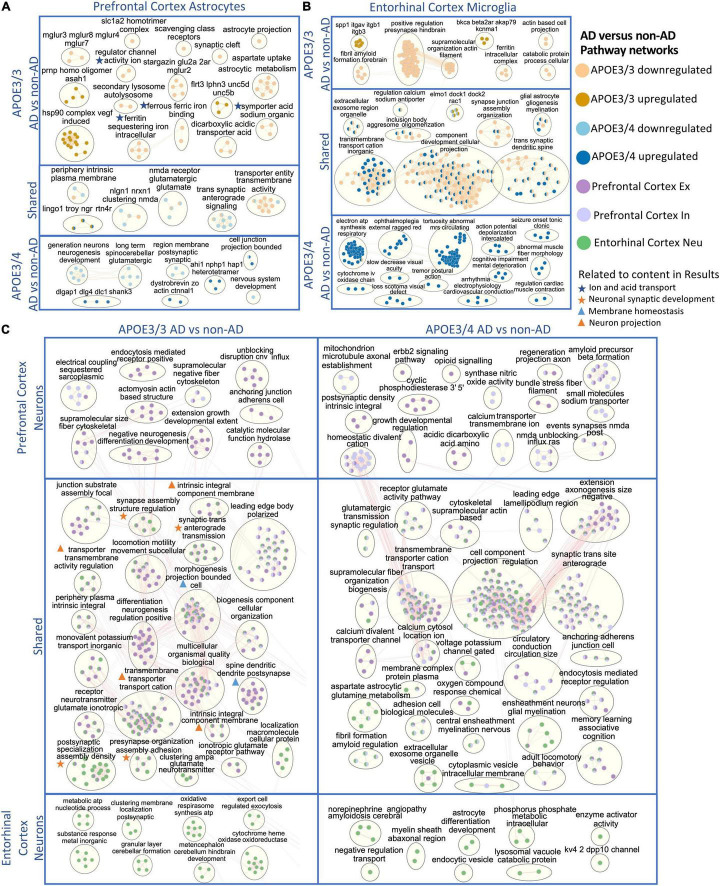
Enriched disease pathway networks in APOE3/3 and APOE3/4 cells. AD compared to non-AD functionally enriched pathways with a BH adjusted *p*-value < 0.01 clustered into biological themes for: **(A)** astrocytes of the prefrontal cortex, **(B)** microglia of the entorhinal cortex, and **(C)** prefrontal cortex excitatory (Ex) and inhibitory (In) neurons, and entorhinal cortex undistinguished neurons (Neu). Lines represent gene set overlaps with magnitude showed by thickness.

Pathway enrichment was performed using g:Profiler (Raudvere et al., 2019), a web tool that performs functional enrichment using an input of gene lists. Separate lists of upregulated and downregulated DEGs in AD relative to control, with a BH corrected adjusted *p*-value < 0.05 and a relaxed expression change cutoff of above 0.1, in each cell type and *APOE* genotype were used as inputs for g:Profiler (Supplementary Tables 6–9). A network analysis was performed to cluster the disease enriched pathways into biologically relevant groups using pathways with an adjusted *p*-value < 0.01 as inputs. Modules of biological themes were generated for each cell type based on the *APOE* genotype (Figure 7 and Supplementary Figures 4, 5).

Through GO enrichment analysis for the prefrontal cortex, we observed pathways related to cell junctions in excitatory neurons, postsynaptic density in inhibitory neurons, Glutamatergic synapse in astrocytes, and Scavenging by Class A Receptors in microglia to be most significantly perturbed in APOE3/3 diseased samples (Supplementary Table 6). In APOE3/4 diseased samples, the top significantly perturbed pathways were related to cell junctions and upregulated in excitatory neurons, inhibitory neurons, and astrocytes, while Apelin signaling pathway was upregulated and most significant in microglia (Supplementary Table 7). Also, we observed terms associated with inflammatory processes such as TNF-α and NF-kappa B signaling complex to be upregulated in APOE3/3 diseased astrocytes compared to controls, but not in APOE3/4. Functional pathways related to synapse organization, neuron differentiation, neurogenesis, and axon development were downregulated in APOE3/4 diseased individuals compared to controls only.

In astrocytes from the prefrontal cortex, we identified six enriched functional modules in both APOE3/3 and APOE3/4 AD relative to controls (Figure 7A). Five out of six were downregulated in AD, and one, the *LINGO1-TROY-NgR* complex, which was previously suggested to be important for modulating glial-neuronal interactions in demyelinating lesions, was upregulated in AD (Satoh et al., 2007). In APOE3/3 astrocytes, ion and acid transport, glutamate receptor activity (mGLUR2, mGLUR3, mGLUR4, mGLUR7, and mGLUR8), metabolic (aspartate uptake and astrocytic metabolism) as well as autolysosome activities (scavenging class receptors, secondary lysosome, and autolysosome) were downregulated in AD, and myelin maintenance (PRNP and ASAH1), cell adhesion (FLRT3, LPHN3, UNC5B, and UNC5D), and Vascular endothelial growth factor (VEGF) induced heat shock protein 90 (hsp90) complex were upregulated in AD, indicating perturbation in processes important for autophagy and stress response which are known to accompany disease progression (Karch and Goate, 2015; Long and Holtzman, 2019). APOE3/4 astrocytes uniquely showed upregulation in pathways related to post-synaptic scaffold proteins (e.g., DLGAP1, DLG4, DLC1, and SHANK3) and actin assembly at cell junctions, but downregulation of synaptic membrane and neurotransmitter pathways, neurogenesis, and nervous system development in AD.

In APOE3/3 astrocytes of the entorhinal cortex, we observed a downregulation of ion and neurotransmitter transport related pathways (intracellular ion and ferritin iron sequestering) in AD. APOE3/4 astrocytes in the entorhinal cortex had mostly upregulated pathway enrichment modules in AD, in contrast to what was observed in prefrontal cortex (Supplementary Figure 5). Many of these pathways governing cellular homeostasis, such as ATP synthesis, transmembrane cation transport, amyloid fibril formation and exosome regulation, and macromolecule and protein plasma membrane localization.

Microglia, the resident brain macrophage, contributes to neuroinflammation in AD and produces APOE upon activation in the brain (Lane et al., 2018; Long and Holtzman, 2019). Differentially enriched pathways were predominantly upregulated in APOE3/4 microglia in AD patients in both prefrontal (Supplementary Figure 4) and entorhinal cortices (Figure 7B), while downregulated in APOE3/3 microglia. Within the entorhinal cortex, changes in gliogenesis, myelination, cation transmembrane transport, cellular projection, synaptic spine development, and synaptic junction assembly pathway network modules were shared in APOE3/3 AD and APOE3/4 AD but perturbed in opposite directions, downregulated in APOE3/3, and upregulated in APOE3/4 microglia (Figure 7B). The *ITGAV-ITGB-SPP1* complex, not previously linked to AD to our knowledge, was significantly upregulated in both brain regions in APOE3/3 microglia in AD, but only in the prefrontal cortex in APOE3/4 microglia in AD (Figure 7B and Supplementary Figure 4). The downregulation of iron homeostasis and ferritin complex, a protein that binds to iron and reflects the level of iron storage in the body, was observed in APOE3/3 microglia and astrocytes of both prefrontal and entorhinal cortex in AD (Figures 7A,B and Supplementary Figure 4).

The GO enrichment analysis of the entorhinal cortex revealed the most pronounce difference between APOE3/3 and APOE3/4 were pathways related to cellular projection development in microglia and astrocytes, such as morphogenesis of plasma membrane, cellular compartment, and plasma membrane bounded cell projection (Supplementary Tables 8, 9). Moreover, GO terms related to homeostatic process, neurogenesis, regulation of transport, and multicellular signaling process were also upregulated in APOE4 all cell types except neurons and downregulated in APOE3/3 microglia and oligodendrocytes.

Overall, network analysis comparing neurons from two brain regions yielded many similar perturbed biological processes within each *APOE* genotype in AD (Figure 7C). In APOE3/3 neurons, shared differentially perturbed processes between brain regions were mostly related to regulation of membrane homeostasis, neuron projection, and synaptic development. Pathway networks in APOE3/3 neurons specific to the prefrontal cortex pertain to cell structure development (actomyosin actin-based structure, extension growth development, anchoring junction, cell adherens), while the entorhinal cortex showed unique modules relevant to cellular energy production (oxidative respirasome synthesis and metabolic ATP nucleotide process). From APOE3/4 neurons, we observed a more diverse population of shared network modules between the two brain regions, including functional processes related to protein trafficking vesicles, myelination, membrane assembly, and voltage gated channel and neurotransmitter receptor regulation. Amyloid fibril formation was uniquely differentially regulated in APOE3/4 neurons and observed in both brain regions in AD, while an amyloid beta precursor formation module was only observed APOE3/4 neurons in prefrontal cortex in AD.

In oligodendrocytes, which provide myelination, APOE3/3 carriers in the prefrontal cortex showed an upregulation of the *ITGAV-ITGB-SPP1* complex and downregulation of pathways related to myelin organization (e.g., juxtaparanode region of axon), ion transport activity, protein refolding, and regulation of MAP kinase signaling activity (e.g., positive regulation of Erk1 and Erk2 in AD). APOE3/4 oligodendrocytes, on the other hand, showed upregulation of stress responses through chaperone mediated protein folding, and downregulation of axon guidance and nervous system development processes in AD. In the entorhinal cortex, we observed modules of processes including neurogenesis, gliogenesis, amyloidosis, aerobic metabolic processes, and exocytosis to be upregulated in APOE3/4 cells and downregulated in APOE3/3 cells in AD (Supplementary Figure 5). Lastly, we observed postsynaptic structural specialization to be uniquely downregulated in APOE3/4 oligodendrocytes.

For OPCs in the prefrontal cortex, there were no common network modules across *APOE* genotypes. In APOE3/3 AD, we identified downregulation for brain cell development processes (*AHI1-NPHP1-HAP1*) (Supplementary Figure 4). In APOE3/4 OPCs, we observed upregulated modules for the ferritin, GAIT and *LINGO1-TROY-NgR* complexes, and downregulation for glutamatergic synaptic activity, plasma membrane and cell organization, and lipoprotein density in AD, which may have implications for neuronal integrity and lipid transport and metabolism. In the entorhinal cortex of AD, we also observed upregulation of the *LINGO1-TROY-NgR*, and downregulation of glutamatergic signaling in APOE3/3 OPCs in AD. Specific to APOE3/4 OPCs in AD, we identified upregulation of processes related to aerobic metabolic processes, stress response, autophagy, amyloid fibril regulation, demyelination, and immune response.

## Discussion

APOE4 is the greatest known genetic risk factor for AD; however, along with other APOE isoforms, its molecular profiles are yet to be investigated at a single-cell level. Here, we analyzed recently available single-cell transcriptomic datasets from two brain regions to better understand how *APOE* genotype plays into transcriptional profiles of AD in a cell type-specific manner. We aimed to understand whether transcriptional differences exist, and if so, how they might be represented in different cell types across brain regions; which cell types were most affected by *APOE* genotype; what changes were shared or dissimilar across cell types; and whether such findings are consistent across brain regions. We performed an *APOE* genotype-stratified differential gene expression analysis comparing AD to control samples within each cell type. Due to the limited number of samples for relatively rare *APOE* genotypes, we restricted our analysis to individuals with APOE3/3 and APOE3/4 genotypes.

In both the prefrontal and entorhinal cortices, we observed shared and unique gene signatures across these *APOE* genotypes that were often cell type-specific, but sometimes spanned many cell types (Figures 2–5). In both brain regions, we observed differing molecular profiles between *APOE* genotypes primarily in glial cells. Interestingly, in both brain regions, we observed a subset of shared DEGs and enriched pathway networks to be perturbed in opposite directions between *APOE* genotypes in AD relative to healthy state, which may indicate potential compensatory or deleterious mechanisms in disease progression in each genotype. Additionally, we observed more DEGs unique to APOE3/4 cells in AD versus control when compared to DEGs for APOE3/3 cells in AD versus control and more DEG overlaps across cell types in APOE3/4 AD, suggesting distinct disease-relevant molecular profiles between *APOE* genotypes and more global AD-related molecular responses when one copy of the *APOE4* allele is present.

In the prefrontal cortex, most DEGs that are common across cell types tend to be more strongly differentially expressed in APOE3/4 AD as compared to those in APOE3/3 AD. Additionally, we observed most of the *APOE* genotype-specific changes in APOE3/4 astrocytes, oligodendrocytes and OPCs, where these genes are predominantly downregulated in AD as compared to controls. Neurons, on the other hand, tended to exhibit DEGs of AD versus control that were common across *APOE* genotypes (Figure 2A and Supplementary Figure 2). Through hierarchical clustering of samples using AD compared to control pseudobulk cell type gene expression (Figure 2C), we observed clustering by *APOE* genotype in all cell types except neurons.

In the entorhinal cortex, microglia and oligodendrocytes had the highest proportion of DEGs of AD versus control that were shared across *APOE* genotypes. Interestingly, these DEGs frequently exhibited opposite changes in expression between APOE3/3 AD cells and APOE3/4 AD cells, implying differing mechanisms of neurodegeneration based on the presence of the APOE4 isoform. Additionally, through hierarchical clustering of samples using AD compared to control pseudobulk cell type gene expression, we observed some influence of brain region and *APOE* genotype on gene expression (Figure 6B). Compared to the prefrontal cortex, the entorhinal cortex, which is implicated in early stages of AD where tau begins to accumulate and the occurrence of synaptic and neuronal loss is associated with the onset of cognitive impairment (Khan et al., 2014; Lane et al., 2018; Long and Holtzman, 2019), had a larger expression change range for DEGs overall, implying a greater magnitude of molecular changes in this region in AD.

Through pathway and network analysis, we identified biological processes potentially involved in AD pathogenesis that were uniquely modified by *APOE* genotype (Figure 7 and Supplementary Figures 4, 5). While many essential cellular processes were differentially regulated in APOE3/3 neurons in AD, most were related to energy production, membrane regulation, and cellular signaling through synapse. APOE3/4 neurons in AD, on the other hand, demonstrated a perturbation of enriched pathways linked to myelination and protein trafficking vesicle regulation (both endocytosis and exosome), which are important cellular processes that protect the integrity of neurons by providing insulation and filtering toxic elements from these cells. This evidence suggests that APOE, a known lipid metabolizing protein, may play differential roles in maintaining essential metabolic processes for neuronal myelination and vesicle trafficking based on its isoform. Glial cells from APOE3/3 and APOE3/4 AD had many uniquely versus common altered biological processes, identified by the *APOE* genotype-specific pathway modules. This suggests that *APOE* genotype modifies glial cell biology in different ways compared to its effects on neuronal cell biology during AD progression. Further study on AD pathogenesis focusing on glial cell modification by the *APOE* genotype might facilitate personalized therapeutic development for AD patients with different *APOE* genotypes.

While we were able to examine *APOE* genotype-specific changes across cell types in both brain regions, some limitations exist. First, due to limited *APOE* genotypes that restricted our analysis to APOE3/3 and APOE3/4 samples, we could not explore more transcriptional profiles such as that of higher AD risk genotypes like APOE4/4. While we focus our analysis on AD-related changes in APOE3/3 and APOE3/4 genotype, it is important to highlight that while one copy of APOE4 can alter gene expression patterns, one copy of APOE3 may be protective against APOE4-related pathological changes. In future studies, it will be of interest to not only include more genotypes such as APOE4/4, but to also compare gene expression changes in associated with APOE genotype in healthy controls.

Each dataset contained only one APOE3/4 control, which was a male sample in both cases. This is a limitation of the data that is currently available and in future studies additional controls should be included. We performed a sensitivity analysis in males of the prefrontal cortex cohort (Supplementary Figure 3), where we also observed more differences in perturbed gene profiles across *APOE* genotypes in astrocytes, oligodendrocytes, and OPCs, and a stronger clustering by *APOE* genotype than cell type identity.

The entorhinal cortex dataset also presents several constraints. It has a small sample size of four cases and five controls, which are also imbalanced with regards to *APOE* genotypes and sex. Specifically, all APOE3/3 samples are from female subjects, all APOE3/4 samples are from male subjects, one of the four cases is an APOE3/3 sample, and one of the five controls is an APOE3/4 sample. Additionally, we observed a batch effect, where cases were sequenced in separate batches from controls, and each batch contained only one sex. To mitigate these limitations, we used Seurat’s integration workflow to integrate the batches and used dimensionality reduction to confirm appropriate batch correction (Supplementary Figure 1). We also included sex as a covariate in our model for differential expression to account for batch while avoiding the collinearity observed with including batch. Another limitation was that the effects of degree of amyloid beta peptide (Aβ) and tau accumulation had not been considered as a potential confounder. These peptides are known to play a major role in Alzheimer disease, with APOE4 increasing accumulation of Aβ and tau neurofibrillary tangles (Schmechel et al., 1993; Shi et al., 2017). Although samples were initially classified as AD or control based on the burden of Aβ plaque and tau neurofibrillary tangles, using CERAD and Braak scores, respectively, a future extension of this work will be to include this potential confounder in the analysis.

Furthermore, we recognize some limits to our findings. Interpretation at the DEG level (Figures 3A, 5A, 6A) was limiting without cell type-specific associations and AD-related mechanistic insights. To consolidate the novel and previously explored DEGs in AD into meaningful insights, we performed a pathway and network enrichment analysis. Comparing disease-relevant signatures across brain regions, we recognize that our observations are influenced by the entorhinal cortex dataset’s constraints and the variability in acquiring each cohort, which is sourced from different sets of individuals and studies. With this limitation, we could not explore further molecular profiles unique to each brain region and their implications for the spread of AD pathology. Overall, the nature of our analysis only allows for association of transcriptomic changes with *APOE* genotype, so links to causality might be hypothesized, but additional followup are needed to prove any such potential links.

Despite the limitations in our study, we present disease-relevant biology with regards to *APOE*-genotype, which we hope spurs further investigation as more single-cell AD datasets become available. We hope that more single-cell AD datasets become available from more brain regions and from diverse sets of individuals, across different ages, racial and ethnic backgrounds, with a greater diversity of *APOE* genotypes and disease severity, thus allowing for more extensive insights. With more diverse genomic data, researchers may (1) integrate datasets from multiple sources and brain regions, (2) examine disease-relevant molecular changes based on *APOE* genotype across brain regions and covariates like age, sex, and severity of pathology, (3) further investigate cell type-and brain region-specificity to uncover *APOE* genotype related molecular profiles associated with the spread pathology, and (4) computationally validate findings with relevant multiomics studies, and subsequently conduct follow-up studies *in vitro* and *in vivo*. Ultimately, we identified key AD-related genes and pathways that are specific to *APOE* genotypes and cell types, especially glial cells, as well as certain consistently affected pathways. These results will inform how glial cells are potentially primary sites of AD-related transcriptional differences based on *APOE* genotype, suggesting possible mechanisms and vulnerable cell subpopulations relevant to AD pathogenesis, and thus can help to facilitate precision medicine diagnostic and drug discovery efforts.

## Data Availability Statement

Publicly available datasets were analyzed in this study. This data can be found here: single nuclei RNA-Seq (snRNA-seq) data and metadata were accessed from their respective repositories: the prefrontal cortex from the Accelerating Medicines Partnership Alzheimer’s Disease Project (AMP-AD) Knowledge Portal under the Religious Orders Study and Memory and Aging Project (ROSMAP) (https://www.synapse.org/#!Synapse:syn18485175 and https://www.synapse.org/#!Synapse:syn3157322), and the entorhinal cortex from a data repository provided by Grubman et al. (2019) (http://adsn.ddnetbio.com/). Data from the entorhinal cortex may also be accessed from the Gene Expression Omnibus under the accession number GSE138852. Access to the prefrontal cortex dataset requires a formal request to ROSMAP. To enable other researchers to explore these datasets, all code necessary for recreating the reported analyses and figures within R, are available on Github at https://github.com/stebel5/AD_APOE_snRNAseq.

## Author Contributions

SB and MS conceived the study. SB designed the study and performed data analysis and interpretation of results (snRNAseq differential gene expression analysis and pathway functional enrichment), generated figures, and drafted the manuscript. DB assisted in developing methods and figures for the analysis and drafted the manuscript. AR assisted in developing methods for data analysis. YL performed network analysis, generated figures for the network analysis, and drafted the manuscript. CS, TO, AT, BG, KZ, GF, and YH contributed to the discussion of methods and results as well as the implications of the findings. MS oversaw the study. All authors read and contributed to the final manuscript.

## Conflict of Interest

YH is a cofounder and scientific advisory board member of Escape Bio, Inc., GABAeron, Inc., and Mederon Bio, LLC. MS is on the advisory board of Aria Pharmaceuticals. The remaining authors declare that the research was conducted in the absence of any commercial or financial relationships that could be construed as a potential conflict of interest.

## Publisher’s Note

All claims expressed in this article are solely those of the authors and do not necessarily represent those of their affiliated organizations, or those of the publisher, the editors and the reviewers. Any product that may be evaluated in this article, or claim that may be made by its manufacturer, is not guaranteed or endorsed by the publisher.

## Footnotes

1
https://www.synapse.org/#!Synapse:syn18485175

2
https://www.synapse.org/#!Synapse:syn3157322

3
http://adsn.ddnetbio.com/

4
https://biorender.com/
