# Supplementary figures and images for: Bioinformatics Analysis of Publicly Available Single-Nuclei Transcriptomics Alzheimer’s Disease Datasets Reveals APOE Genotype-Specific Changes Across Cell Types in Two Brain Regions
Source: Front Aging Neurosci. 2022 Apr 27;14:749991. doi: 10.3389/fnagi.2022.749991 (PMC9093608; doi:10.3389/fnagi.2022.749991)

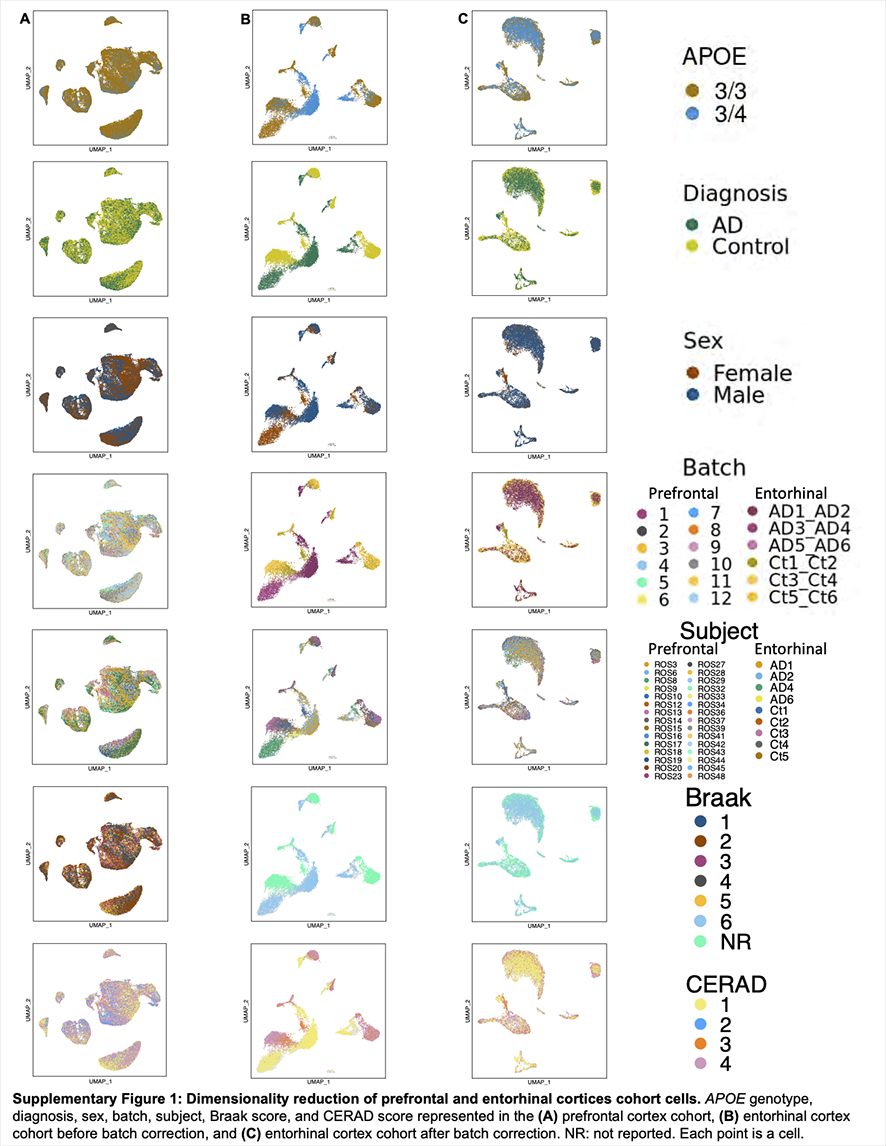

Supplement: Supplementary file 2 [file Image_1.TIFF]

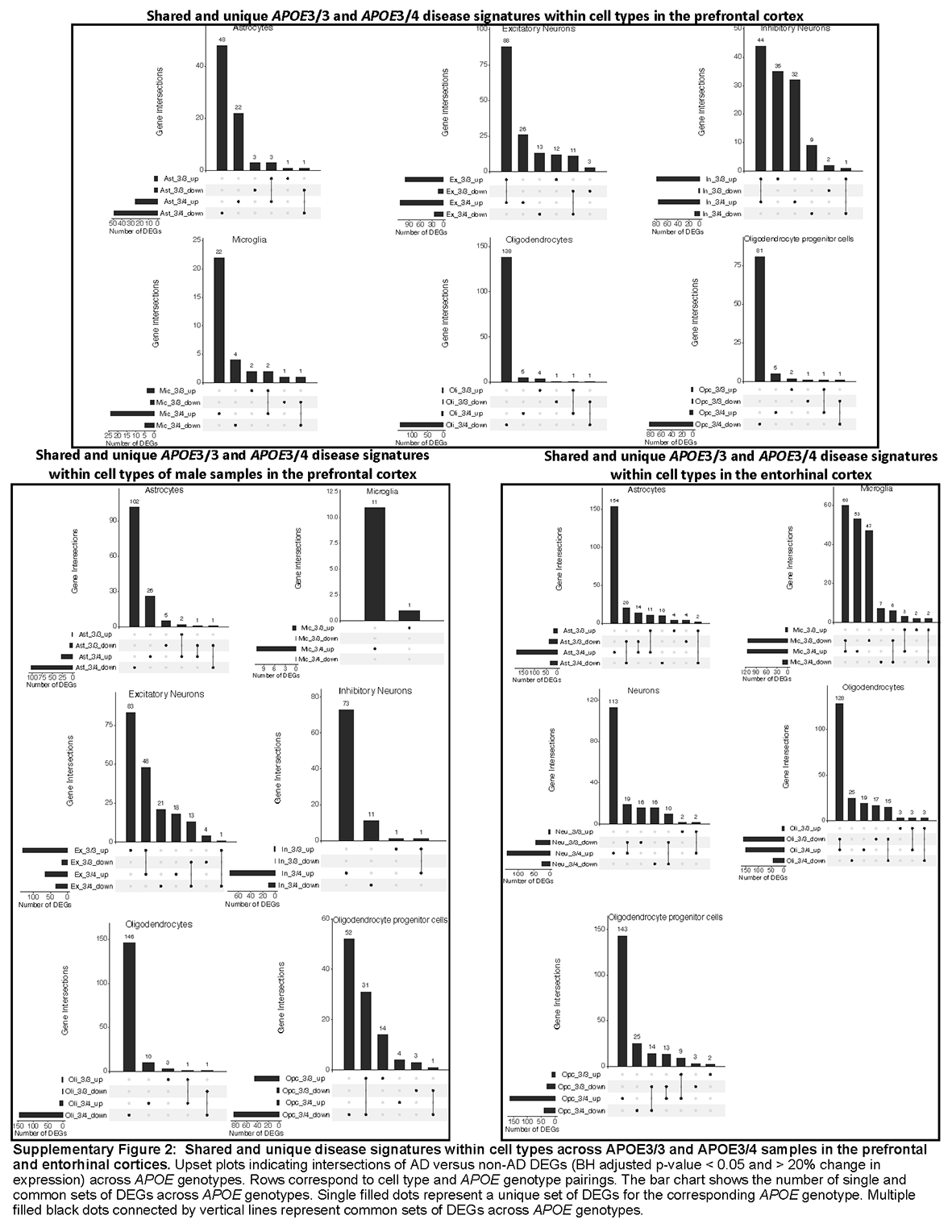

Supplement: Supplementary file 3 [file Image_2.TIFF]

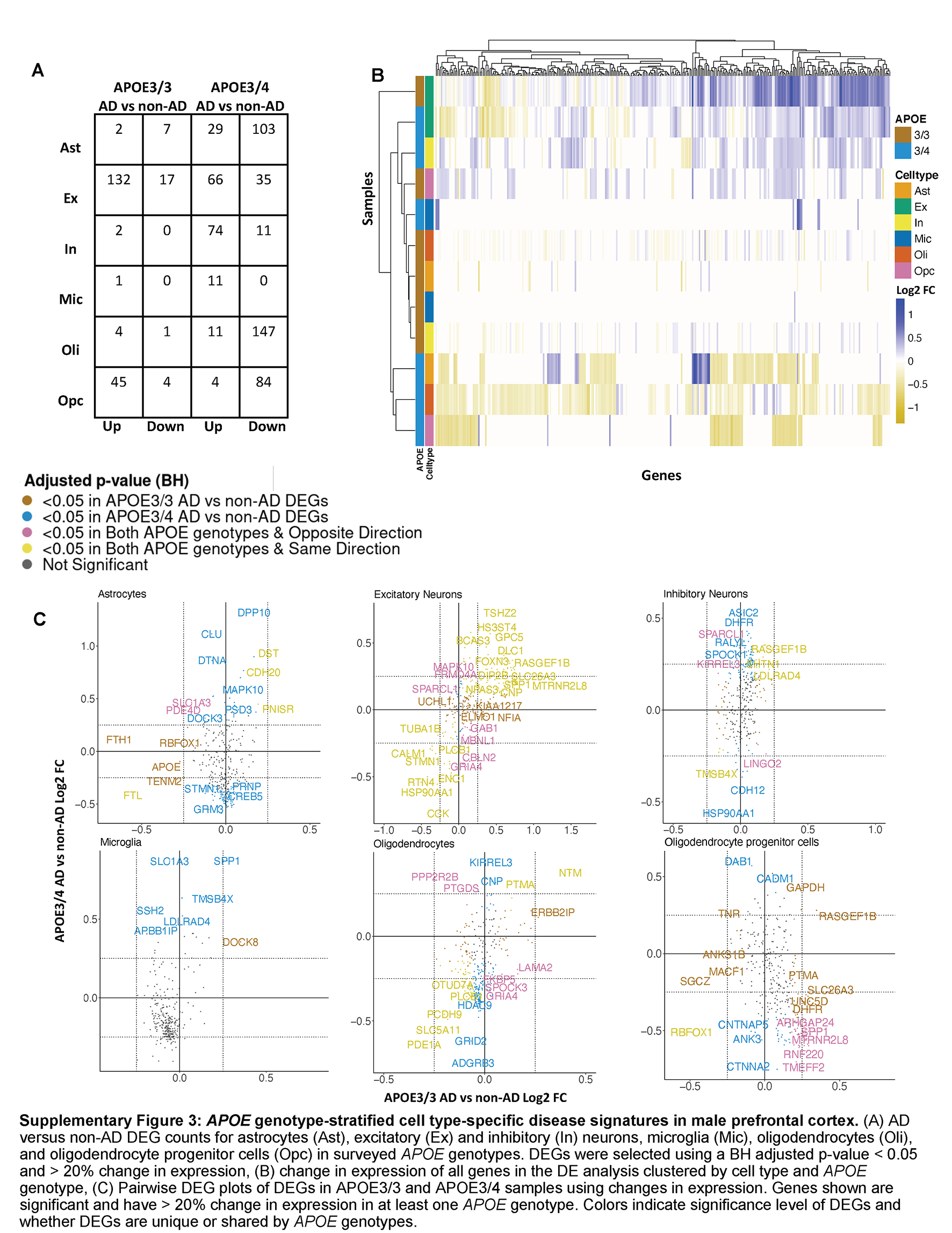

Supplement: Supplementary file 4 [file Image_3.TIFF]

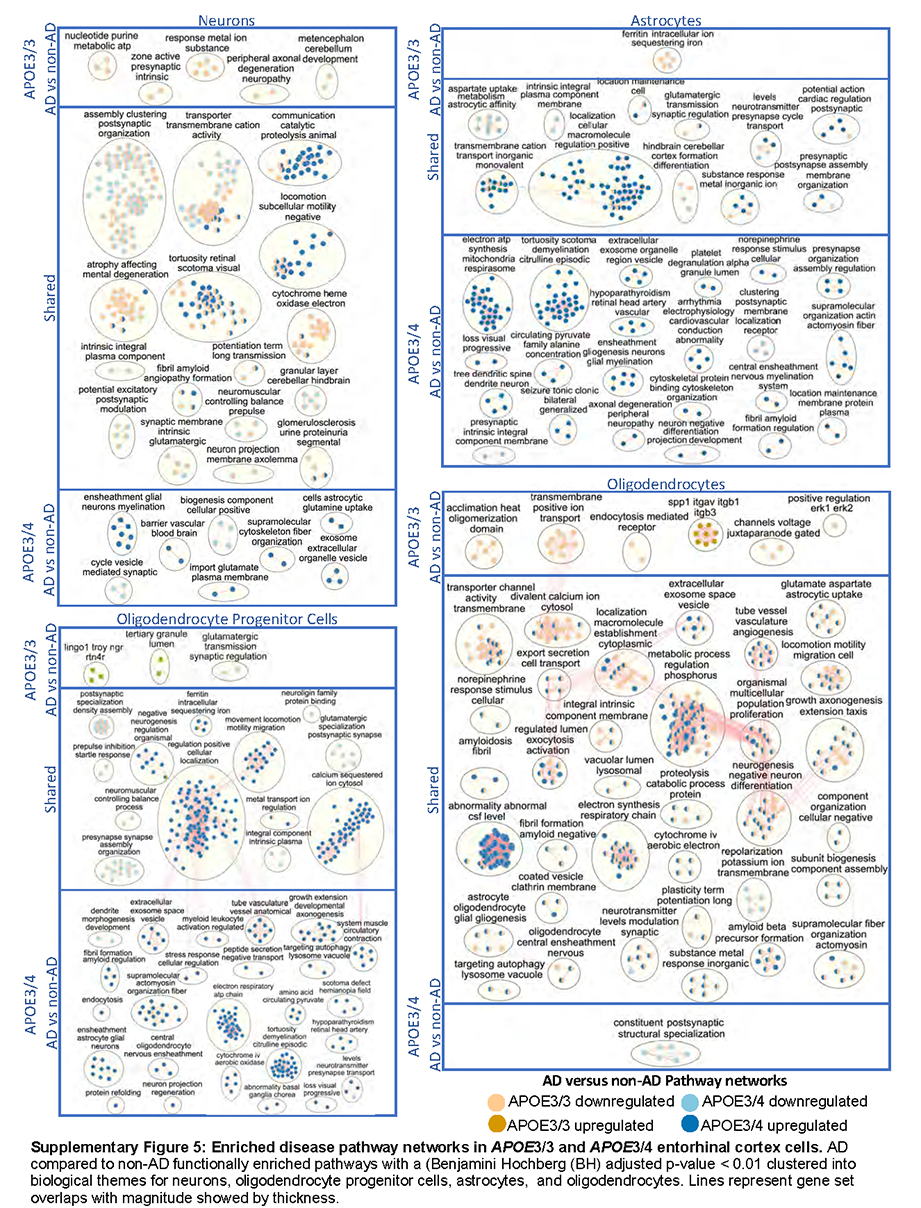

Supplement: Supplementary file 6 [file Image_5.TIFF]
